# Interpersonal sensitivity mediates the effects of perceived parenting styles on state anxiety and negative assessment of life events in adult volunteers from the community

**DOI:** 10.1002/pcn5.98

**Published:** 2023-05-18

**Authors:** Tomoteru Seki, Chihiro Morishita, Yoshitaka Ishii, Ayaka Deguchi, Motoki Higashiyama, Yoshio Iwata, Miki Ono, Mina Honyashiki, Jiro Masuya, Ichiro Kusumi, Takeshi Inoue

**Affiliations:** ^1^ Department of Psychiatry Tokyo Medical University Tokyo Japan; ^2^ Department of Psychiatry Hokkaido University Graduate School of Medicine Hokkaido Japan

**Keywords:** care, multiple regression analysis, overprotection, parenting style, structural equation model

## Abstract

**Aim:**

The association of parenting experiences in childhood with anxiety symptoms in adulthood has yet to be clarified. We hypothesized that interpersonal sensitivity (IPS) mediates the impacts of parenting experiences in childhood on anxiety symptoms and negative assessment of life events in adulthood.

**Methods:**

An observational cross‐sectional study was carried out from January 2014 to August 2014 on 853 adults. Participants provided their demographic information and answered the following four self‐administered questionnaires: Parental Bonding Instrument (PBI), Interpersonal Sensitivity Measure (IPSM), Life Experiences Survey (LES), and State–Trait Anxiety Inventory Form Y (STAI‐Y). The data of a total of 404 participants who agreed to take part in this study were analyzed.

**Results:**

Multiple regression analysis with the State Anxiety subscale of STAI‐Y as the dependent variable identified the following five out of the 15 independent variables as being statistically significant: IPSM total, LES positive and negative, PBI paternal overprotection, and employment status. This model explains 17.8% of the State Anxiety subscale score. In the structural equation models, the Care subscale showed significant indirect negative effects on State Anxiety subscale and LES negative score through a decrease in IPSM total score (*β* = –0.061 and –0.042, respectively). The former indirect effect accounted for 31.6%, and the latter accounted for 56.8% of the total effects. In contrast, Overprotection subscale had opposite effects to Care subscale.

**Conclusion:**

These results suggest that parenting experiences in childhood are related to adult anxiety symptoms and the negative assessment of life events indirectly through IPS.

## Graphical abstract

In this study, we investigated the effects of the perceived parenting style in childhood, which is classified into care and overprotection, on state anxiety through interpersonal sensitivity and the assessment of life‐changing events. This study suggests that perceived negative parenting experiences in childhood are associated with adult state anxiety and the negative assessment of life events indirectly through interpersonal sensitivity.

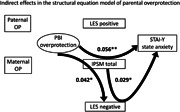

## INTRODUCTION

Anxiety is a response to unknown, internal, and vague threats.^1^ Anxiety often occurs in people with depressive disorders.^2^, ^3^ Disorders involving anxiety, such as major depressive disorder^4^, ^5^ and anxiety disorders, such as panic disorder,^2^, ^5^ generalized anxiety disorder,^2^ and obsessive compulsive disorder,^5^, ^6^ have been reported to adversely influence the quality of life.

Parenting styles are methods of raising children, which include elements of control and supervision of the children's behavior and actions, support and care, other specific attitudes, and behavior patterns that influence children's outcomes.^7^ Perceived parenting styles reflect the bonds between the children and caregivers, and are closely related to child development.^8^ Parents should provide a suitable environment for children to develop self‐esteem, self‐confidence, self‐control, and the ability to develop meaningful relationships with others outside the family circle.^9^ Parker et al.^8^ suggested that the combination of high care and low overprotection might be conceptualized as optimal. In contrast, negative/dysfunctional parenting styles (e.g., “affectionless control”) are considered to be closely associated with the future development of anxiety sensitivity,^10^ and moreover, adult mental health disorders.^11^ Specifically, negative parenting styles experienced in childhood have been considered to act as risk factors for anxiety symptoms,^10^ and to be associated with adult depression,^12^ including postnatal depression.^13^, ^14^, ^15^ The combination of a low level of care, which denotes indifference and rejection, and high overprotection, which denotes control and intrusion, has been referred to as affectionless control parenting.^8^ Patients with depression^11^, ^12^, ^16^, ^17^ and anxiety disorders^11^, ^17^, ^18^, ^19^ often perceive that they experienced this parenting style in childhood. However, there is a time lag between perceived parenting style in childhood and mental health in adulthood, and thus it is unlikely that perceived parenting style itself has a direct effect on mental health in adulthood. Regarding maltreatment, which is a factor associated with negative parenting styles, the mediating role of interpersonal sensitivity (IPS) in the influence of child abuse on adult anxiety was reported by Nakazawa et al.^20^ However, no studies to date have shown that IPS mediates the impacts of perceived parenting styles on state anxiety in adulthood.

IPS is defined as an “undue and excessive awareness of and sensitivity to the behavior and feelings of others, particularly criticism or rejection,” and is a personality trait that is considered to be a premorbid personality of depression.^21^ Previously, minimal attention was paid to the association between IPS and anxiety disorders, but several studies have recently reported the association between IPS and anxiety disorders.^22^, ^23^ Moreover, a study targeting subjects with an at‐risk mental state (ARMS) reported higher IPS in individuals with an ARMS compared with controls, and found that IPS was associated with positive psychotic symptoms in addition to symptoms of depression and anxiety.^24^ Harb et al.^22^ reported that a group of patients with social anxiety disorder had higher IPS than controls. The association between IPS and anxiety disorders has also been reported by Wilhelm et al.^23^ We previously reported that IPS had a direct positive effect on state anxiety.^20^ Furthermore, we found that the impact of IPS on state anxiety was mediated by evaluating life events negatively.^20^

Several associations between assessing life events negatively and depression, anxiety, and tension have also been reported. Yasuda et al.^25^ reported a positive association between negative change score in life events and state anxiety. In other words, those who assessed life events more negatively felt greater anxiety. Uchida et al. reported that there was a positive correlation between the negative appraisal of life events and state anxiety.^26^ Using the same sample as the study of Uchida et al., Nakazawa et al.^20^ similarly reported that the negative evaluation of life events showed a significant direct positive effect on state anxiety. In a recent study, Lei et al. found that only subjects in the group with high depression and high anxiety levels had experienced more negative life events in the previous 6 months, or 7 months to 1 year previously than subjects in the healthiest group.^27^

Attachment is the primary bond that each individual infant forms with one of its caregivers (usually the mother).^7^ Bowlby viewed attachment as a product of evolutionary processes,^28^ that is, infants are born with an innate drive to seek interaction and form attachments with parents in order to survive. Gradually and repeatedly, children interpret and react to the parents' response when approaching the parents.^29^ The attachment theory proposes that the internalization of attachment experiences through interaction with parents in childhood will form working models.^30^, ^31^, ^32^, ^33^ Working models formed in early childhood persist stably throughout life, and routinely influence actions associated with interpersonal relationships.^30^, ^31^, ^32^ During early childhood, affectionless control by parents inhibits the formation of a positive working model (representational model), and subjects who experience this type of dysfunctional parenting tend to have interpersonal conflicts and increased susceptibility to depressive and anxiety disorders following stress.^34^ Wilhelm et al. reported that fragile inner‐self, which is a subscale of IPS, is associated with low care and high overprotection, particularly from the mother.^23^ A previous study by Otani et al.^35^ demonstrated that high parental overprotection, particularly affectionless control^36^ from a parent of the same sex, increases IPS. Zhang et al.^37^ reported that both maternal and paternal overprotection are associated with IPS, whereas maternal rejection had a greater impact on IPS than paternal rejection.

It has also been pointed out that particular personality traits influence the subjective assessment of life events. Although there have only been a few studies to date, interpersonal relationships have also been reported to be associated with the subjective evaluation of life experiences.^38^ In addition, our previous study showed that IPS was positively correlated with the negative evaluation of life events.^20^

The objective of this study was to clarify the association between the parenting style experienced in childhood and anxiety symptoms in adulthood. We hypothesized that IPS mediates the long‐term impacts of perceived parenting styles on adult state anxiety and the negative assessment of life events. In addition, the indirect effect of IPS on state anxiety via the negative evaluation of life events has already been reported,^20^ and the same effect was also expected to be observed in this study.

## METHODS

### Subjects

In 2014, written self‐administered questionnaires were distributed to 853 Japanese adult volunteers from the community for a larger study.^20^, ^26^ The present study designed as cross‐sectional belongs to a larger study, and the subjects were informed that participation in this research was voluntary. We anonymized the collected information so that the individuals could not be identified. The inclusion criterion for this study was being 20 years of age or older. The exclusion criteria were having a serious physical disease that has a significant impact on mental health (e.g., “malignant tumor”) and having an organic brain syndrome. The questionnaires were returned from 455 subjects (53.3%) to the research group by mail, and 51 of these subjects were excluded from this study owing to many missing values on their questionnaires. A total of 404 subjects who gave their consent to participate in this study and provided demographic information and answered the four questionnaires, as shown below, were analyzed. This study was approved by the Institutional Review Committees of Tokyo Medical University and Hokkaido University (study approval no.: SH3308 and 010‐0041, respectively), in compliance with the Declaration of Helsinki (2013).

### Questionnaires

#### Parental Bonding Instrument

The Parental Bonding Instrument (PBI) assesses how adult subjects retrospectively recall their parents' nurturing attitudes that they experienced until the age of 16 years. PBI was developed by Parker et al.,^8^ and the Japanese version of the PBI was developed and validated by Kitamura and Suzuki.^39^ PBI is composed of 25 items, including two subscales; that is, Care (e.g., “Spoke to me with a warm and friendly voice”) and Overprotection (e.g., “Tried to control everything I did”). Respondents answer each of them on a 4‐point Likert scale (0–3). The higher the total Care score, the stronger subjects felt affection, warmth, empathy, and closeness from their parents. On the other hand, the higher the total score of Overprotection, the stronger the subjects felt that their parents had controlled them and had not respected or encouraged their independence. PBI was performed for each paternal nurturing attitude and maternal nurturing attitude. Paternal and maternal scores for Care and Overprotection were separately used in the analysis. In addition, two latent variables, referred to as “Care” and “Overprotection,” composed of paternal and maternal scores were used in the structural equation models.

#### Interpersonal Sensitivity Measure

The Interpersonal Sensitivity Measure (IPSM) measures an individual's sensitivity to others. IPSM was developed by Boyce and Parker,^21^ and the Japanese version of IPSM was developed by Kuwabara et al.^40^ IPSM consists of 36 items, which are categorized into five subscales; that is, Interpersonal Awareness (e.g., “I worry about the effects I have on other people”), Need for Approval (e.g., “I will go out of my way to please someone I am close to”), Separation Anxiety (e.g., “I feel insecure when I say goodbye to people”), Timidity (e.g., “I will do something I do not want to do rather than offend or upset someone”), and Fragile Inner‐Self (e.g., “My value as a person depends enormously on what others think of me”). Respondents answer each of them on a 4‐point Likert scale (1–4). High scores indicate greater interpersonal sensitivity. The total score of all items was used in the analysis.

#### Life Experiences Survey

The Life Experiences Survey (LES) assesses whether subjects experienced various life events in the previous year. If subjects experienced life events, then they rate the intensity of their psychological impacts. LES was developed by Sarason et al.,^41^ and the Japanese version was developed and validated by our research group.^42^ Respondents answer each of 57 items of life events (e.g., “Marriage”) on a 7‐point scale (–3 to 3 points). Each positive and negative change score is calculated, by summing scores rated as positive or negative by the subjects, respectively. Higher change scores, whether positive or negative, indicate a greater intensity of their psychological impacts in that direction. Both negative change scores and positive change scores were used in this study.

#### State–Trait Anxiety Inventory Form Y

The State–Trait Anxiety Inventory Form Y (STAI‐Y) separately evaluates trait anxiety and state anxiety. STAI‐Y was developed by Spielberger et al.,^43^ and the Japanese version was developed by Hidano et al.^44^ This study used State Anxiety scores consisting of 20 items (e.g., “I am tense”). Higher scores indicate higher state anxiety. Respondents answered each item on a 4‐point Likert scale (1–4).

### Statistical analysis

For estimating the associations of demographic information (sex, employment status, marital status, current physical disease, and first‐degree relative with psychiatric disease) with STAI‐Y State Anxiety score, the *t*‐test was performed. The group statistics of demographic information (sex, employment status, marital status, current physical disease, and first‐degree relative with psychiatric disease) were presented as numbers with means ± SD of STAI‐Y State Anxiety score.

For estimating the correlations of demographic information (age, education years, number of offspring) and the results of the self‐administered questionnaires with STAI‐Y State Anxiety score, Pearson correlation coefficients were calculated. Absolute values of the Pearson correlation coefficients represent the strength of the correlation, which can be described to be small (around 0.1), moderate (around 0.3), and large (around 0.5).^45^ Positive and negative Pearson correlation coefficients represent the direction of correlation. The descriptive statistics of demographic information (age, education years, and number of offspring), and scores of PBI, IPSM, LES, and STAI‐Y were presented as means ± SD.

For eliminating possible confounding factors that might be associated with state anxiety, stepwise multiple regression analysis was performed with state anxiety as the dependent variable and the following 15 participant characteristics or scores as the independent variables: age, sex, education years, employment status, marital status, number of offspring, current physical disease, psychiatric disease in a first‐degree relative, PBI (care and overprotection from the father and the mother), total IPSM score, and LES (positive and negative scores). The square of the multiple correlation coefficient (*R* ^2^) was used to evaluate how well the independent variables can explain the variation of a dependent variable. The *R* ^2^ value for the multiple regression analysis represents the strength of the total regression, which can be described to be small (around 0.02), moderate (around 0.13), and large (around 0.26).^45^ The variance inflation factor (VIF) was used to evaluate multicollinearity. A VIF greater than 10 indicates multicollinearity. Standardized partial regression coefficients (beta) were calculated from standardized variables, and represent the strength and the direction of the associations between the variables. The absolute value of beta represents the strength of the association. Positive and negative beta values represent the direction of association from independent variables to a dependent variable. SPSS 28 software (IBM) was used for the estimations and analysis of the above.

**Figure 1:**
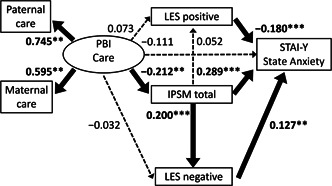
The results of direct effects in the structural equation model with parental care, interpersonal sensitivity, assessment of life events, and state anxiety in 404 adult volunteers from the community. Rectangles represent observed variables, and the oval represents the latent variable. Solid arrows represent statistically significant pathways, and dotted arrows represent nonsignificant pathways. The numbers beside the arrows represent the direct standardized coefficients. **p < 0.01, ***p < 0.001. IPSM, Interpersonal Sensitivity Measure; LES, Life Experiences Survey; PBI, Parental Bonding Instrument; STAI‐Y, State–Trait Anxiety Inventory Y.

**Figure 2:**
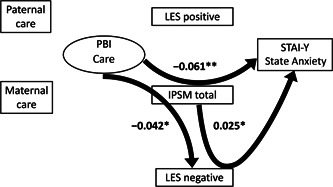
The results of indirect effects in the structural equation model with parental care, interpersonal sensitivity, assessment of life events, and state anxiety in 404 adult volunteers from the community. Rectangles represent observed variables, and the oval represents the latent variable. Solid arrows represent statistically significant pathways, and the nonsignificant indirect pathways are not shown. The numbers beside the arrows represent the indirect standardized coefficients. *p < 0.05, **p < 0.01. IPSM, Interpersonal Sensitivity Measure; LES, Life Experiences Survey; PBI, Parental Bonding Instrument; STAI‐Y, State–Trait Anxiety Inventory Y.

**Figure 3:**
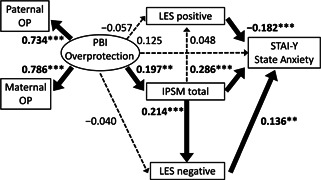
The results of direct effects in the structural equation model with parental overprotection, interpersonal sensitivity, assessment of life events, and state anxiety in 404 adult volunteers from the community. Rectangles represent observed variables, and the oval represents the latent variable. Solid arrows represent statistically significant pathways, and dotted arrows represent nonsignificant pathways. The numbers beside the arrows represent the direct standardized coefficients. **p < 0.01, ***p < 0.001. IPSM, Interpersonal Sensitivity Measure; LES, Life Experiences Survey; OP, overprotection; PBI, Parental Bonding Instrument; STAI‐Y, State–Trait Anxiety Inventory Y.

**Figure 4:**
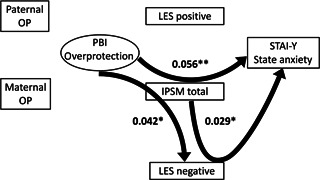
The results of indirect effects in the structural equation model with parental overprotection, interpersonal sensitivity, assessment of life events, and state anxiety in 404 adult volunteers from the community. Rectangles represent observed variables, and the oval represents the latent variable. Solid arrows represent statistically significant pathways, and the nonsignificant indirect pathways are not shown. The numbers beside the arrows represent the indirect standardized coefficients. *p < 0.05, **p < 0.01. IPSM, Interpersonal Sensitivity Measure; LES, Life Experiences Survey; OP, Overprotection; PBI, Parental Bonding Instrument; STAI‐Y, State–Trait Anxiety Inventory Y.

For finding indirect effects from parenting styles that were overlooked because they were found to be insignificant in multiple regression analysis, structural equation models were built using Mplus Version 8.5 software (Muthén & Muthén). In the structural equation models, two fit indices, namely, the comparative fit index (CFI) and root‐mean‐square error of approximation (RMSEA), were used to evaluate the fit of the model. A CFI greater than 0.95 and an RMSEA less than 0.08 indicate an acceptable fit; and a CFI greater than 0.97 and an RMSEA less than 0.05 indicate a good fit.^46^ The *R* ^2^ value was used to evaluate how well this model explains the variability. All coefficients of the covariance structure analysis were standardized. The standardized coefficients (*β*) were calculated from standardized variables, and represent the strength and the direction of the associations between the variables. Based on the assumed chronological order from the hypothesis described in the Introduction section, structural equation models for the Care subscale of the PBI (Figures 1 and 2) and for Overprotection (Figures 3 and 4) were built, using IPSM total score, LES positive and negative scores, and State Anxiety score of STAI‐Y. The design of the study has been described in detail in our previous study.^47^ A *p*‐value of less than 0.05 was considered to indicate a statistical significance between groups.

## RESULTS

### Association or correlation of participant characteristics and each measure with STAI‐Y State Anxiety subscale

Participant characteristics, scores of PBI, IPSM, LES, and STAI‐Y, and their associations or correlations with STAI‐Y State Anxiety score are shown in Tables 1 and 2. Demographic data did not have an association or were not correlated with State Anxiety score. PBI scores for the Care subscale (care from the father and the mother) were negatively correlated with State Anxiety score (*r* = −0.141, *p* = 0.004 and *r* = −0.122, *p* = 0.014, respectively), and PBI scores for the Overprotection subscale (overprotection from the father and the mother) were positively correlated with State Anxiety score (*r* = 0.170, *p* < 0.001, and *r* = 0.128, *p* = 0.010, respectively). IPSM total score was positively correlated with State Anxiety score (*r* = 0.333, *p* < 0.001), and LES positive and negative scores were negatively and positively correlated with State Anxiety score, respectively (*r* = −0.160, *p* = 0.001, and *r* = 0.171, *p* < 0.001, respectively).

**Table 1:** Participant characteristics and their associations with STAI‐Y State Anxiety score in adult volunteers.

Characteristic	Value (number)	Association with STAI‐Y score (mean ± SD)
Sex (men:women)	220:184	Men (40.4 ± 10.1) versus women (40.0 ± 10.5), *p* = 0.713
Employment status (unemployed:employed)	56:341	Unemployed (37.8 ± 11.1) versus employed (40.6 ± 10.2), *p* = 0.057
Marital status (unmarried:married)	114:287	Unmarried (41.5 ± 10.4) versus married (39.8 ± 10.2), *p* = 0.131
Current physical disease (no:yes)	319:81	No (40.0 ± 10.4) versus yes (41.3 ± 10.1), *p* = 0.291
First‐degree relative with psychiatric disease (no:yes)	362:40	No (40.1 ± 10.3) versus yes (41.8 ± 10.9), *p* = 0.318

*Note*: Data of the group statistics in the *t*‐test are presented as numbers with means ± SD.

Abbreviations: SD, standard deviation; STAI‐Y, State–Trait Anxiety Inventory Y.

**Table 2:** Participant characteristics, and scores of PBI, IPSM, LES, and STAI‐Y and their correlations with STAI‐Y State Anxiety score in adult volunteers.

Characteristic or measure	Value (mean ± SD)	Correlation with STAI‐Y score (*r*)
Age (years)	42.3 ± 11.9	*r* = 0.000, *p* = 1.000
Education years	15.2 ± 2.0	*r* = −0.010, *p* = 0.847
Number of offspring	1.3 ± 1.2	*r* = 0.048, *p* = 0.333
PBI		
Paternal care	23.9 ± 7.2	*r* = −0.141, *p* = 0.004**
Maternal care	27.7 ± 6.7	*r* = −0.122, *p* = 0.014*
Paternal overprotection	9.5 ± 6.1	*r* = 0.170, *p* < 0.001***
Maternal overprotection	10.2 ± 6.8	*r* = 0.128, *p* = 0.010*
IPSM total	84.5 ± 14.2	*r* = 0.333, *p* < 0.001***
LES		
Positive	1.7 ± 3.0	*r* = −0.160, *p* = 0.001**
Negative	1.7 ± 3.1	*r* = 0.171, *p* < 0.001***
STAI‐Y state anxiety	40.3 ± 10.3	

*Note*: Data are presented as means ± SD. *r*, Pearson correlation coefficient.

Abbreviations: IPSM, Interpersonal Sensitivity Measure; LES, Life Experiences Survey; PBI, Parental Bonding Instrument; SD, standard deviation; STAI‐Y, State Trait Anxiety Inventory Form Y.

* *p* < 0.05

** *p* < 0.01

*** *p* < 0.001.

### Stepwise multiple regression analysis of the State Anxiety subscale of STAI‐Y

Table 3 shows the results of stepwise multiple regression analysis with the State Anxiety subscale of STAI‐Y as the dependent variable. The stepwise method identified the following five out of the 15 independent variables as being statistically significant: IPSM total, LES positive and negative, PBI paternal overprotection, and employment status. The adjusted version of the *R* ^2^ was 0.178. Variance inflation factors were from 1.021 to 1.086, and multicollinearity was denied.

**Table 3:** Statistically significant variables identified by stepwise multiple regression analysis of STAI‐Y State Anxiety score.

Independent variable	Unstandardized coefficient	Standardized coefficient	*p*‐value	95% CI for B		VIF
	B	Beta		Lower	Upper	
IPSM total	0.227	0.316	<0.001	0.159	0.296	1.086
LES positive	−0.615	−0.179	<0.001	−0.932	−0.298	1.021
PBI paternal overprotection	0.241	0.142	0.003	0.084	0.398	1.023
LES negative	0.396	0.118	0.014	0.079	0.712	1.067
Employment status (unemployed = 0, employed = 1)	3.413	0.114	0.015	0.658	6.168	1.022
Adjusted *R* ^2^ = 0.178	*F* = 17.532, *p* < 0.001					

Abbreviations: B, unstandardized coefficient; Beta, standardized partial regression coefficient; CI, confidence interval; F, ratio of the mean square regression divided by the mean square error; IPSM, Interpersonal Sensitivity Measure; LES, Life Experiences Survey; PBI, Parental Bonding Instrument; *R* ^2^, square of the multiple correlation coefficient; STAI‐Y, State Trait Anxiety Inventory Form Y; VIF, variance inflation factor.

### Analysis of the structural equation model of parental care

In the model of Figures 1 and 2, the fit indices were regarded as an acceptable fit (CFI = 0.964 and RMSEA = 0.054). β scores from PBI Care were 0.745 for the father and 0.595 for the mother, meaning that the contribution of paternal care was stronger than that of maternal care. The *R* ^2^ of STAI‐Y State Anxiety in this model was 0.173, that is, this model explains 17.3% of the variability in STAI‐Y State Anxiety scores of adult volunteers.

#### Direct effects

Five significant direct effects were observed (Figure 1). IPSM total and LES negative and positive had direct effects on State Anxiety (*β* = 0.289, *p* < 0.001, *β* = 0.127, *p* = 0.006, and *β* = –0.180, *p* < 0.001, respectively). PBI Care had a direct effect on IPSM total (*β* = –0.212, *p* = 0.002), and IPSM total had a direct impact on LES negative (*β* = 0.200, *p* < 0.001).

#### Indirect effects

Three out of the nine indirect impacts were significant (Figure 2). IPSM total mediated the effects of PBI Care on State Anxiety (*β* = –0.061, *p* = 0.003) and LES negative (*β* = –0.042, *p* = 0.019). The former indirect effect accounted for 31.6%, and the latter for 56.8% of the total effects, respectively. LES negative mediated 8.2% of the total impact of IPSM total on State Anxiety (*β* = 0.025, *p* = 0.039). The following six indirect impacts (not shown in Figure 2) were not significant: the mediating role of LES positive and negative on the effect of PBI Care on State Anxiety (*β* = –0.013, *p* = 0.440 and *β* = –0.004, *p* = 0.772, respectively), the mediating role of IPSM total with LES positive and negative in the effect of the PBI Care on State Anxiety (*β* = 0.002, *p* = 0.390 and *β* = –0.005, *p* = 0.092, respectively), the mediating role of IPSM total on the effect of PBI Care on LES positive (*β* = –0.011, *p* = 0.392), and the mediating role of LES positive on the effect of IPSM total on State Anxiety (*β* = –0.009, *p* = 0.358).

### Analysis of the structural equation model of parental overprotection

In the model of Figures 3 and 4, the fit indices indicated a good fit (CFI = 0.983 and RMSEA = 0.046). *β* scores from PBI Overprotection were 0.734 for the father and 0.786 for the mother. The *R* ^2^ of STAI‐Y State Anxiety in this model was 0.177, that is, this model explains 17.7% of the variability in STAI‐Y State Anxiety scores of adult volunteers.

#### Direct effects

There were five significant direct impacts (Figure 3). IPSM total and LES negative and positive had direct effects on State Anxiety (*β* = 0.286, *p* < 0.001, *β* = 0.136, *p* = 0.004, and *β* = –0.182, *p* < 0.001, respectively). PBI Overprotection had a direct impact on IPSM total (*β* = 0.197, *p* = 0.001), and IPSM total had a direct impact on LES negative (*β* = 0.214, *p* < 0.001).

#### Indirect effects

Three out of the nine indirect impacts were significant (Figure 4). IPSM total mediated the impacts of PBI Overprotection on State Anxiety (*β* = 0.056, *p* = 0.002) and LES negative (*β* = 0.042, *p* = 0.014). The former indirect impact accounted for 29.3% of the total impact, whereas the percentage of the latter indirect impact was incalculable. LES negative mediated 9.5% of the total impact of IPSM total on State Anxiety (*β* = 0.029, *p* = 0.033). Six of the indirect effects (not shown in Figure 4) were not significant; that is, the mediating role of LES positive and negative on the effect of PBI Overprotection on State Anxiety (*β* = 0.010, *p* = 0.344 and *β* = –0.005, *p* = 0.600, respectively), the mediating role of IPSM total with each LES positive and negative in the effect of PBI Overprotection on State Anxiety (*β* = –0.002, *p* = 0.392 and *β* = 0.006, *p* = 0.084, respectively), the mediating role of IPSM total in the effect of PBI Overprotection on LES positive (*β* = 0.009, *p* = 0.387), and the mediating role of LES positive in the effect of IPSM total on State Anxiety (*β* = –0.009, *p* = 0.376).

## DISCUSSION

We found that care measured on the Care subscale of the PBI did not directly but indirectly decreased state anxiety in the STAI‐Y through a decrease in IPSM total score. On the other hand, overprotection had opposite effects to care. Previous studies have already clarified the associations between perceived parenting styles and IPS,^23^, ^35^, ^36^, ^37^ and between IPS and state anxiety,^20^ respectively. However, how the style of parenting experienced in childhood is related to state anxiety in adulthood through IPS has not been reported to date. To the best of our knowledge, this is the first study to suggest that parenting experiences in childhood do not directly, but indirectly affect state anxiety in adulthood through the effect on IPS, using covariance structure analysis on adult volunteers. This is also the first study to suggest that IPS mediates the effects of parenting experiences in childhood on the negative assessment of live events. Interestingly, although child abuse had a statistically significant direct positive impact on state anxiety besides an indirect impact via IPS in the previous study by Nakazawa et al.,^20^ neither overprotection nor care had a direct impact on state anxiety in this study. Even though the experience of maltreatment in childhood and negative parenting experiences in childhood are similar regarding the time gap to the development of adult anxiety, why childhood maltreatment has a direct impact on state anxiety in adulthood whereas negative parenting experiences in childhood do not remains unclear, and further research is required in the future.

The results of previous studies^23^, ^35^, ^36^, ^37^ are consistent with those of the present study, suggesting that parental overprotection and low care enhance IPS. IPS is one of the main psychological problems that affects these subjects from their adolescence. In a recent study on adolescents,^37^ both maternal and paternal overprotection were found to be associated with IPS. In the same study, paternal emotional warmth was negatively correlated with IPS, but the correlation was not found for maternal emotional warmth in ordinary least squares linear regression.^37^ The contributions of the mother and the father to the effect of overprotection on IPS were similar between this study and the study by Zhang et al.^37^ In addition, the fact that the father's influence was stronger than that of the mother regarding the effect of care on IPS in this study was consistent with the sex difference in the association between emotional warmth and IPS in the study of Zhang et al.^37^

Several studies demonstrating that the correlation between parenting styles and particular personality traits correlated with IPS^47^, ^48^, ^49^ also possibly indirectly support the results of our present study. For example, Furukawa reported that maternal overprotection and neuroticism were positively correlated in both male and female 17‐ to 19‐year‐old university students.^48^ We have also reported the association between negative parenting styles and neuroticism.^47^, ^49^ On the other hand, there is another study that reported that each subscale of IPSM consistently correlates with neuroticism, therefore suggesting that IPS is a personality trait associated with neuroticism.^21^, ^23^, ^50^ Based on these studies, studies that show the association between negative parenting style and neuroticism may indirectly support the association between negative parenting style and IPS. Regarding childhood maltreatment, which occurs in similar contexts as negative parenting styles, our previous studies by Otsuka et al.^51^ and Nakazawa et al.^20^ reported that the impacts of childhood maltreatment enhance IPS. These studies also enabled us to predict the results of this study to some extent.

Our previous study using the same subjects as this study already showed that IPS enhances the negative assessment of life events and has an indirect effect on state anxiety through the negative assessment of life events.^20^ However, there are still few studies showing the association between IPS and the subjective assessment of life events. Focusing on neuroticism associated with IPS, several studies have reported the association between neuroticism and adult life events. For example, Yasuda et al.^25^ performed correlation analysis and reported a positive correlation between neuroticism measured by the Neo Five Factor Inventory and negative change scores in life events measured by the LES. Furthermore, we reported in our previous study by Ono et al.^47^ that neuroticism enhanced the negative assessment of life events.

Our present study suggests that support for parents and education for improving dysfunctional parenting may reduce IPS in children during adolescence to adulthood, and then reduce state anxiety after reaching adulthood. Consequently, it may lead to a reduction in medical expenses. Based on the results of this study, intervention of IPS itself may also reduce state anxiety both directly and indirectly through improving the negative assessment of life events. As IPS is a personality trait that affects people at the pre‐adult stage, early intervention of IPS is desirable. Psychotherapy focused on IPS would be useful for moderating IPS. For example, interpersonal psychotherapy is a short‐term psychotherapy focusing on patients' interpersonal problems or their emotions in association with their interpersonal problems, which leads to improvement of their depressive symptoms. Interpersonal psychotherapy has also been used for a range of other conditions, including social anxiety disorder, and has demonstrated to be effectiveness.^52^ However, as the correlation coefficients in our structural equation models were generally weak, and these models explained only 17% of the variability in state anxiety, the impacts of parenting and IPS on state anxiety are seen to a limited extent in the population of this study. Other influencing factors on state anxiety should be considered together for establishing actual psychological interventions.

### Limitations

This study had some limitations. First, although we built structural equation models considering the chronological order of experiences in life (i.e., perceived parenting styles in childhood, IPS in adolescence to adulthood, assessment of life events experienced in the previous year, and anxiety symptoms after becoming adults), as this is a cross‐sectional study, no conclusions about the causal associations between variables can be made. Second, as we recruited subjects by convenience sampling, it is possible that there is sampling bias, and the subjects might not represent the general population. Third, the results may be biased, because the data were collected using a self‐completed questionnaire. Finally, there may also be recall bias as subjects were looking back on their childhood experiences from many years previously.

## CONCLUSION

Our study suggested that parenting experiences in childhood indirectly affect state anxiety and the negative assessment of life events through their effects on IPS, and identified intervenable variables for improving state anxiety. Large‐scale prospective studies are required in the future to verify that IPS has mediating roles on the impacts of perceived parenting styles on anxiety.

## AUTHOR CONTRIBUTIONS

All authors made a significant contribution to the work reported, whether that is in the conception, study design, execution, acquisition of data, analysis and interpretation, or in all these areas; took part in drafting, revising or critically reviewing the article; gave final approval of the version to be published; have agreed on the journal to which the article has been submitted; and agree to be accountable for all aspects of the work.

## FUNDING INFORMATION

This work was partly supported by a Grant‐in‐Aid for Scientific Research (no. 21K07510 to T. Inoue) from the Ministry of Education, Culture, Sports, Science and Technology‐Japan.

## CONFLICT OF INTEREST STATEMENT

Jiro Masuya has received personal compensation from Otsuka Pharmaceutical, Eli Lilly, Astellas, and Meiji Yasuda Mental Health Foundation, and grants from Pfizer. Ichiro Kusumi has received personal compensation from Astellas, Chugai Pharmaceutical, Daiichi Sankyo, Dainippon Sumitomo Pharma, Eisai, Eli Lilly, Janssen Pharmaceutical, Kyowa Hakko Kirin, Meiji Seika Pharma, MSD, Nippon Chemiphar, Novartis Pharma, Ono Pharmaceutical, Otsuka Pharmaceutical, Pfizer, Tanabe Mitsubishi Pharma, Shionogi, and Yoshitomiyakuhin, and has received research grants from AbbVie GK, Asahi Kasei Pharma, Astellas, Boehringer Ingelheim, Chugai Pharmaceutical, Daiichi Sankyo, Dainippon Sumitomo Pharma, Eisai, Eli Lilly, GlaxoSmithKline, Kyowa Hakko Kirin, Meiji Seika Pharma, MSD, Novartis Pharma, Ono Pharmaceutical, Otsuka Pharmaceutical, Pfizer, Takeda Pharmaceutical, Tanabe Mitsubishi Pharma, Shionogi, and Yoshitomiyakuhin, and is a member of the advisory board of Dainippon Sumitomo Pharma and Tanabe Mitsubishi Pharma. Takeshi Inoue has received personal compensation from Mochida Pharmaceutical, Takeda Pharmaceutical, Eli Lilly, Janssen Pharmaceutical, MSD, Taisho Toyama Pharmaceutical, Yoshitomiyakuhin, and Daiichi Sankyo; grants from Shionogi, Astellas, Tsumura, and Eisai; and grants and personal compensation from Otsuka Pharmaceutical, Dainippon Sumitomo Pharma, Mitsubishi Tanabe Pharma, Kyowa Pharmaceutical Industry, Pfizer, Novartis Pharma, and Meiji Seika Pharma; and is a member of the advisory boards of Pfizer, Novartis Pharma, and Mitsubishi Tanabe Pharma. The other authors declare that they have no actual or potential conflicts of interest associated with this study.

## ETHICS APPROVAL STATEMENT

This study was approved by the Institutional Review Committees of Tokyo Medical University and Hokkaido University (study approval no.: SH3308 and 010‐0041, respectively), in compliance with the Declaration of Helsinki (2013).

## PATIENT CONSENT STATEMENT

All subjects were informed that participation in this research was voluntary and the collected information was anonymized so that the individuals could not be identified. Only the subjects who gave their consent to participate in this study were analyzed.

## CLINICAL TRIAL REGISTRATION

N/A.

## Data Availability

The data supporting the findings of this study are available from the corresponding author, upon reasonable request.
